# A framework for the design and analysis of phase III randomised trials in uncommon diseases

**DOI:** 10.1186/1745-6215-16-S2-O56

**Published:** 2015-11-16

**Authors:** Mahesh Parmar, Tim Morris, Matthew Sydes

**Affiliations:** 1 MRC Clinical Trials Unit at UCL, London, UK

## 

There is a methodological gap between common diseases, where large trials can be undertaken, and truly rare diseases, where increasingly trial designers perform a complete paradigm shift and use Bayesian methods to design and analyse a trial. A key issue for these ‘uncommon diseases’ is that it can be infeasible to recruit enough patients in a reasonable timeframe using traditional frequentist trial design parameters. However, there remains a pressing need to improve treatments in these situations. We present a framework for designing trials to fill the gap between common and rare diseases.

Staying with the frequentist approaches used in large trials that are well understood and accepted, we propose several alterations to the design parameters. When we put these together, they provide important reductions to the required sample size. The parameters we consider are: the targeted effect size, type I error rate, power and skewing the allocation ratio. Other aspects we consider are: choosing appropriate outcome measures, leveraging covariate information, re-randomising participants and borrowing external information. We discuss the benefits of some of these changes and warn against others, and consider the consequences of making a wrong decision. We illustrate, with a worked example, the savings that can be achieved without requiring a complete move away from the paradigm that is established and widely accepted in more common diseases. This framework should allow us to improve outcomes for patients with uncommon diseases through conducting rigorous phase III randomised trials.

